# Impact of pleural integrity during mammary artery harvesting on short term outcome

**DOI:** 10.1186/1749-8090-8-S1-O120

**Published:** 2013-09-11

**Authors:** M Pano, S Zaccaria, A Scotto di Quacquaro, G Floris, D Rocco, P Fellini, G Scrascia, P Pelini, M Cucurachi

**Affiliations:** 1 Department of Cardiac Surgery, “Vito-Fazzi” Hospital, Lecce, Italy; 2 Department of Cardiac Anesthesia, “Vito-Fazzi” Hospital, Lecce, Italy

## Background

The aim of this retrospective study was to evaluate if the impact of pleural integrity during left internal mammary artery (LIMA) harvesting might influence short term outcome.

## Methods

From May 2012 to May 2013, 136 patients undergoing isolated CABG operation (with or without pump) were enrolled in the study. The mammary artery was always harvested in a skeletonized fashion. These patients were split in two groups: Group A (96 pts) with open pleura and Group B (40 pts) with intact pleura. The two groups were comparable regarding pre- and operative data.

## Results

There were no differences in mean values between Group A and Group B for: Age, BMI, LVEF, CPB time, Cross-clamp time, Number of anastomoses / patients. Group A and Group B were significantly different in terms of ventilation time ( 13,47+/-18,2 h vs 8,4+/-6,3h, p<0,001), bleeding within 12h (540,31+/-283,5 ml vs 392,25+/- 257,8ml, p<0,001), blood transfusion units (1,38+/-1,6 vs 0,6+/-1,4, p<0,001), and length of hospital stay ( 14,7+/-14,3 day vs 11,2+/-7, p<0,001).

## Conclusions

Our data showed that preservation of pleura integrity, when possible, during LIMA harvesting has a strong impact on post-operative course. Pleural integrity can reduce postoperative bleeding with a minor need of blood transfusion. Very likely these finding along with a less time of ventilation might reduce the length of hospital stay.

